# Systemic lupus erythematosus with trisomy X: a case report and review of the literature

**DOI:** 10.1186/s13256-022-03478-5

**Published:** 2022-07-19

**Authors:** Fang Luo, Qiao Ye, Jie Shen

**Affiliations:** Department of Rheumatology, The Second Affiliate Hospital of Jiaxing University, Jiaxing, 314000 China

**Keywords:** Systemic lupus erythematosus, Trisomy X, Female

## Abstract

**Background:**

The cause of systemic lupus erythematosus is not completely clear so far, but the prevalence of systemic lupus erythematosus is significantly increased in people with additional X chromosomes.

**Case presentation:**

We report a 17-year-old Chinese female patient with systemic lupus erythematosus complicated with trisomy X, accompanied by lupus nephritis, pancytopenia, hemolytic anemia, and multiserous effusion. The patient recovered well after treatment and returned regularly. We review the previously reported cases to summarize the clinical characteristics of these patients.

**Conclusion:**

The additional X chromosome is related to the development of systemic lupus erythematosus. Whether it is a subtype of systemic lupus erythematosus remains to be further confirmed.

## Introduction

Systemic lupus erythematosus (SLE) is an autoimmune disease with circulating immune complexes deposition and multiorgan involvement. It predominantly affects females, especially women of childbearing age, with a female-to-male ratio of 9:1 [1]. Although the gender bias has often been attributed to sex hormones, the risk of developing SLE in men with an extra X chromosome is similar to that in 46,XX normal women and a 14-fold higher risk of developing SLE than 46,XY males, while female patients with Turner’s syndrome (45,X0) abstained from contracting SLE [2, 3]. In addition, the prevalence of SLE in 47,XXX women is approximately 2.5 times higher than that in 46,XX women [4]. It suggests X chromosomes may contribute to the susceptibility of human SLE. SLE in females with extra X chromosomes have been described in several cases.

Here, we present a case of a 17-year-old girl who developed SLE with 47,XXX, and review similar literature to discuss the relationship between X chromosomes and SLE.

## Case presentation

A 17-year-old Chinese girl with no previous medical history or particular family history was admitted to our hospital with fever, headache, and weakness for 4 days. The patient developed paroxysmal headache and dizziness at school, followed by low-grade fever and weakness, without cough and expectoration. She then went to the emergency room of another hospital for treatment. During her hospitalization, she developed abdominal pain and computed tomography scan of the chest and abdomen revealed multiple lymphadenopathy and serous cavity effusion. After treatment with ceftriaxone and oseltamivir, the symptoms were not relieved, and her temperature peaked at 38.9 °C. Subsequently, the patient was admitted to our hospital for further diagnosis and treatment.

Physical examination at admission revealed a fever of 37.7 ℃, anemic, abdominal tenderness, and anterior tibial edema. Several enlarged lymph nodes with slight tenderness were palpable in her bilateral neck and armpit. The patient’s height and weight were 168 cm and 48 kg, respectively, and her expression was indifferent.

Laboratory findings revealed elevated acute phase reactants with erythrocyte sedimentation rate (ESR) at 112 mm, and C-reactive protein (CRP) at 213.5 mg/L. Blood tests revealed pancytopenia and anemia (total white blood cell count: 2.27 × 10^9^/L; hemoglobin level: 65 g/L; red blood cells 1.81 × 10^9^/L, platelets 89 × 10^9^/L; reticulocyte 1.9%). Urine tests showed proteinuria (1.66 g/days) and serum albumin was 26.5/L. Coombs test was positive and platelet antibody test was negative. The immunoglobulin G (IgG) was 24.5 g/dL and complement (C3 and C4) levels were decreased. Anticardiolipin antibody IgG was positive. The patient tested positive for the following autoantibodies: high titer of antinuclear antibody of 1:1000, positivity for anti-double-stranded DNA (dsDNA) antibody, anti-nRNP/Sm antibody, anti-SS-A/Ro and anti-SS-B/La antibodies, anti-histone antibody, anti-ribosomal P protein antibody, and anti-nucleosome antibody. Tumor series and blood culture were normal.

Ultrasonography showed bilateral pleural effusion. Magnetic resonance of the brain revealed a few small cavities in the left semi-soft circle area and bilateral frontal cortex. No organic lesions were found on enhanced computed tomography scan of small intestine. Low bone mineral density detected by the dual-energy X-ray absorptiometry (lumbar spine: Z-score, 1.7).

**Fig. 1:**
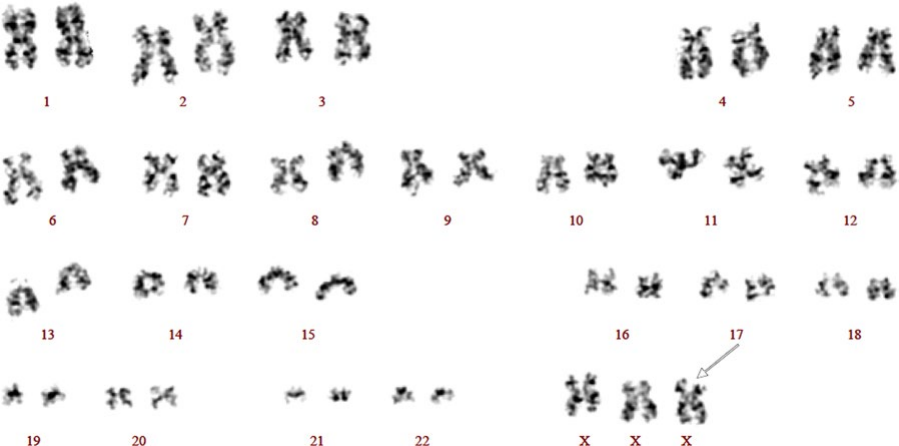
Karyotype of this patient

To rule out other hematologic diseases, bone marrow examination was performed, which showed no malignancy, but the Giemsa banding revealed a karyotype of 47,XXX in all 20 metaphase cells analyzed (Figure 1).

The patient was diagnosed with SLE on the basis of fever, hemolytic anemia, pancytopenia, low C3 and C4, proteinuria greater than 0.5 g/day, serous effusion, antinuclear antibody positive, and anti-dsDNA positive, fulfilling the 2019 European League Against Rheumatism/American College of Rheumatology (EULAR/ACR) criteria [5], complicated with lupus nephritis, pancytopenia, hemolytic anemia, serous effusion, lacunar cerebral infarction, and trisomy X syndrome. Although she had positive anti-SS-A/Ro and anti-SS-B/La antibodies, and anticardiolipin antibody, she did not fulfill the classification criteria of Sjögren’s syndrome (SS) or antiphospholipid syndrome. The SLE disease activity index (SLEDAI) score was 17, indicating the patient had high disease activity. The patient was treated successfully with intravenous gamma globulin at 17.5 g/day for 5 days to block antibodies, methylprednisolone 240 mg/day for 3 days (then the dose of methylprednisolone was gradually reduced), oral hydroxychloroquine 0.2 g/day, along with transfusion of washed red blood cells, albumin infusion, therapy of calcium supplement, stomach protection, diuresis, and anticoagulation. The symptoms were relieved after 2 weeks of treatment, edema disappeared, and ultrasonography did not show bilateral pleural effusion. Because of the lupus nephritis, the patient was treated with intravenous cyclophosphamide 0.6 g per 2 weeks. When the cumulative dose of cyclophosphamide reached 3 g, the blood cell count, serum albumin, C-reactive protein, and erythrocyte sedimentation rate were normal, anticardiolipin antibody IgG was negative, and urinary protein decreased to 0.44 g/day. Considering the patient has not given birth and the inhibitory effect of cyclophosphamide on gonads, we decided to discontinue the treatment of cyclophosphamide and start with belimumab as a remission-maintenance drug. After altering to belimumab, the patient's condition remained stable.

## Discussion

Trisomy X syndrome (47,XXX) is characterized by the presence of an additional X chromosome, which presents in 1 of every 1000 live-born females [6]. Its incidence is related to increased age of the mother. Physical findings described in women with 47,XXX are the presence of epicanthal folds, hypertelorism, upslanted palpebral fissures, clinodactyly, overlapping digits, pes planus, pectus excavatum, hypotonia, and joint hyperextensibility. The extra chromosome may cause premature ovarian failure, and proportion of infertility is slightly higher than that of normal people [7]. The patient in our case presented menarche at the age of 15 years and had normal menstrual cycles. Some patients with trisomy X syndrome may have delayed development and personality disorder, but most patients with trisomy X syndrome have mild or asymptomatic symptoms. In this case, the patient’s hypertelorism and indifference may be related to trisomy X syndrome. However, she had no difficulty in learning and communication.

We summarized the clinical characteristics of patients with SLE with trisomy X syndrome [8–10] (Table 1). All patients presented with anemia, of which five cases were hemolytic anemia. In addition, three cases had arthritis and four cases had lupus nephritis. The results suggest that lupus nephritis, arthritis, and hemolytic anemia may be common clinical features of SLE in 47,XXX women. Among them, four patients tested positive for anti-RNP and anti-SSA/SSB antibodies, but there were no related clinical manifestations. In addition, the age of onset of these patients was relatively young, including three cases with diagnosis for childhood-onset systemic lupus erythematosus (cSLE).

**Table 1:** Present and reported cases of SLE in female with trisomy X

	Reported cases					Present case
	Case 1	Case 2	Case 3	Case 4	Case 5	
Year of report	1987	1991	2016	2020	2021	2021
Age at onset of SLE (years)	26	21	28	11	16	17
Ethnicity	Caucasian	Asian	Asian	African	Asian	Asian
Clinical manifestations						
Hematologic disorder	Hemolytic anemia lymphopenia	Hemolytic anemia	Hemolytic anemia	Hemolytic anemia	Anemia pancytopenia	Hemolytic anemia pancytopenia
Neurological disorder	ND	Epilepsy	(−)	(−)	(−)	(−)
Renal disorder	ND	(+)MPGN	(−)	(+)NS	(+)No biopsy	(+)No biopsy
Serositis	ND	ND	(−)	(−)	(−)	(+)
Joint manifestations	Polyarthritis	ND	Arthritis	Arthritis	(−)	(−)
Mucocutaneous manifestation	ND	ND	(−)	(−)	Malar erythema oral ulcer	(−)
Laboratory index						
Antinuclear antibody	(+)	(+)	(+)	(+)	(+)	(+)
Anti-DNA antibody	ND	(+)	(+)	(+)	(+)	(+)
Anti-Sm antibody	ND	ND	(−)	ND	(+)	(+)
Anti-SS-A/B antibody	(+)	ND	(+)	ND	(+)	(+)
Anti-RNP antibody	(+)	ND	(+)	ND	(+)	(+)
Low complement	(−)	(+)	(+)	(+)	(+)	(+)

ND, not described; MPGN, mesangioproliferative glomerulonephritis; NS, nephrotic syndrome

SLE and 47,XXX both occur in 1 in 1000 women [11]; if the conditions were independent, only 1 in 1 million would have both SLE and 47,XXX. However, an increased incidence of 47,XXX in cSLE was observed. In another study involving 2826 patients with SLE, 7 were women with 47,XXX, with the prevalence of SLE with 47,XXX being about 2.5 times higher than that of 46,XX women [4]. Slae M *et al*. performed a systemic literature review for cases of polysomy X and SLE and summarized previously published cases, which showed that X chromosome polysomy may confer increased susceptibility to SLE [12]. Sharma *et al*. examined cohorts of patients with SS and SLE by constructing intensity plots of X chromosome single-nucleotide polymorphism alleles, along with determining the karyotype of selected patients. The results showed that 3 patients with a triple mosaic, consisting of 45,X/46,XX/47,XXX among ~ 2500 women with SLE, and 1 patient had 45,X/46,XX/47,XXX among ~ 2100 women with Sjögren’s syndrome(SS), and neither the triple mosaic nor the partial triplication were found among the controls. Thus, it indicated X-chromosome abnormalities were present among patients with either SS or SLE and may inform the location of genes that mediate an X dose effect [13]. The previous data suggested the number of X chromosomes was responsible for SLE in men [14].

The mechanism between abnormal X chromosome and the risk of SLE remains unclear. At the stage of female embryo development, one X chromosome is inactivated randomly, but this inactivation is not complete. About 15% of the genes encoded by X chromosomes can escape inactivation [15]. Some microRNAs (miRNAs) encoded by X chromosome were already suggested as being involved in the pathogenesis of SLE. The expression of miR-106a is regulated by *EGR1* and *Sp1*, and disordered expression of miR-106a leads to the pathogenesis of SLE [16]. Overexpression of miR-224 inhibits the protein expression of api5, which can promote cell death induced by T-cell activation in SLE [17]. In addition, downregulation of miR-98 may regulate the expression of Fas-mediated apoptosis, signaling pathway in CD4+T cells, which induces apoptosis death in patients with SLE [18]. Some genes related to SLE are located on the X chromosome; therefore, asymmetric X inactivation and chimerism can induce the pathogenesis of SLE. In addition, demethylation of CD40LG, a B cell costimulatory molecule encoded on the X chromosome, may lead to reactivation of inactive X chromosome, which contributes to SLE [19] and provides another possible explanation for the gene dose hypothesis.

Several genes on X chromosome have been confirmed to be associated with SLE in humans. *IRAK1* and its adjacent gene *MECP2*, located at Xq28, are related genes for pathogenesis of SLE [20]. Three potential signals (rs2071128 in *NAA10*, rs5987175 in *LCA10*, and rs17422 in *TMEM187*) are related to the risk of SLE [21]. A meta-analysis of two genome-wide association studies (GWAS) regarding SLE in Chinese Han population identified a novel variant in *PRPS2* on Xp22.3 as associated with SLE [22]. Other genes located on X chromosome, such as *TLR7* (Xp22.2), *TLR8* (Xp22.2), and *OGT* (Xq13.1) have been implicated in the development of SLE. Many immunity-related genes are located in X chromosome, which are overexpressed and cause diseases. As a result, humans with extra X chromosomes may suffer higher risk of developing SLE.

Most patients described in Table 1 are Asian, while other studies have proved that patients with SLE with 47,XXX are mainly European [4]. However, owing to selection bias and other factors, the results reported cannot reflect the real situation. Therefore, the relationship between ethnicity and the incidence of 47,XXX needs to be further studied. In other immune system diseases, such as SS, the incidence of 47,XXX was increased, while the prevalence of 47 XXX in primary biliary cirrhosis (PBC) and rheumatoid arthritis(RA) did not increase significantly [4]. Therefore, the difference in the incidence of 47,XXX in different immune diseases needs to be further studied.

It is reported that patients with SLE complicated with trisomy X may have a higher risk of ischemic necrosis and osteoporosis, which may be related to the long-term use of steroids, premature ovarian failure, and overexpression of FVII on X chromosome [10]. In addition, trisomy X syndrome has a high risk of infertility and SS. For this patient, we should pay attention to patient’s fertility and the risk of developing SS and bone complications in the follow-up.

## Conclusion

This case report reveals that additional X chromosome is related to the development of SLE. However, SLE combined with 47,XXX is relatively rare in women, but whether it is a subtype of SLE remains to be further confirmed, and the exact mechanisms of X-chromosome dosage in SLE remain unknown.

## Data Availability

Not applicable.
